# Engaging communication experts in a Delphi process to identify patient behaviors that could enhance communication in medical encounters

**DOI:** 10.1186/1472-6963-10-97

**Published:** 2010-04-19

**Authors:** Jaya K Rao, Lynda A Anderson, Bhuvana Sukumar, Danielle A Beauchesne, Terry Stein, Richard M Frankel

**Affiliations:** 1 Division of Pharmaceutical Outcomes and Policy, Eshelman School of Pharmacy, University of North Carolina at Chapel Hill, 2202 Kerr Hall, CB 7573, Chapel Hill, NC 27599, USA; 2 Healthy Aging Program, Centers for Disease Control and Prevention 4770 Buford Highway NE, MS K-45, Atlanta, GA 30341, USA; 3 ICF Macro International Inc, 3 Corporate Square, Suite 370, Atlanta, GA 30329, USA; 4 The Permanente Medical Group, Kaiser Permanente Northern California, Oakland, CA 94612, USA; 5 Center for Implementing Evidence Based Practice, Roudebush VA Medical Center, 1481 West 10thStreet, Indianapolis, Indiana, 46202, USA

## Abstract

**Background:**

The communication literature currently focuses primarily on improving physicians' verbal and non-verbal behaviors during the medical interview. The Four Habits Model is a teaching and research framework for physician communication that is based on evidence linking specific communication behaviors with processes and outcomes of care. The Model conceptualizes basic communication tasks as "Habits" and describes the sequence of physician communication behaviors during the clinical encounter associated with improved outcomes. Using the Four Habits Model as a starting point, we asked communication experts to identify the verbal communication behaviors of patients that are important in outpatient encounters.

**Methods:**

We conducted a 4-round Delphi process with 17 international experts in communication research, medical education, and health care delivery. All rounds were conducted via the internet. In round 1, experts reviewed a list of proposed patient verbal communication behaviors within the Four Habits Model framework. The proposed patient verbal communication behaviors were identified based on a review of the communication literature. The experts could: approve the proposed list; add new behaviors; or modify behaviors. In rounds 2, 3, and 4, they rated each behavior for its fit (agree or disagree) with a particular habit. After each round, we calculated the percent agreement for each behavior and provided these data in the next round. Behaviors receiving more than 70% of experts' votes (either agree or disagree) were considered as achieving consensus.

**Results:**

Of the 14 originally-proposed patient verbal communication behaviors, the experts modified all but 2, and they added 20 behaviors to the Model in round 1. In round 2, they were presented with 59 behaviors and 14 options to remove specific behaviors for rating. After 3 rounds of rating, the experts retained 22 behaviors. This set included behaviors such as asking questions, expressing preferences, and summarizing information.

**Conclusion:**

The process identified communication tasks and verbal communication behaviors for patients similar to those outlined for physicians in the Four Habits Model. This represents an important step in building a single model that can be applied to teaching patients and physicians the communication skills associated with improved satisfaction and positive outcomes of care.

## Background

Patient-physician communication is recognized as an important aspect of health care quality and patient safety [1,2]. The number of investigations examining strategies to enhance physician-patient communication is growing. For example, a recent systematic review identified 36 randomized controlled trials of interventions designed to enhance physician or patient communication behaviors [3]. These interventions generally resulted in improved communication behaviors among physicians and patients. In particular, physicians in the intervention groups often received higher ratings of their overall communication style and exhibited specific patient-centered communication behaviors more often than those in the control groups. Similarly, intervention patients obtained more information from their physicians and exhibited greater involvement during visits than controls [3].

Although prior investigations have demonstrated substantial progress in improving specific communicative behaviors of physicians and patients, they have tended to focus exclusively on either the physician or patient, and do not examine how the physician's and patient's communication behaviors relate to each other during the interaction. These limitations were noted by Inui and colleagues in 1985 [4], and to a certain extent, may result from the lack of an overarching framework that describes the sequence of physician and patient communication behaviors as they might occur during the course of a clinical visit.

The Four Habits Model addresses these concerns by focusing on how temporal and sequential elements of the encounter relate to each another and to outcomes (Table 1) [5-9]. This framework identifies the basic communication tasks of the clinical encounter and conceptualizes how these tasks or "habits" relate to one another during the visit. The four habits include: Invest in the beginning (Habit 1), Elicit the patient's perspective (Habit 2), Demonstrate empathy (Habit 3), and Invest in the end (Habit 4). Each habit includes a group of physician communication behaviors and skills that are associated with effective clinical practice and positive health outcomes [7,8].

**Table 1:** The Four Habits Model

Habit	Skills	Techniques & Examples			Pay-off		
**Invest in the ** **beginning**	Create rapport quickly	• Introduce self to everyone in the room • Acknowledge wait • Convey knowledge of patient's history by commenting on prior visit or problem • Attend to patient's comfort • Make a social comment or ask a non-medical question to put patient at ease • Adapt own language, pace, and posture in response to patient			• Establishes a welcoming atmosphere • Allows faster access to real reason for visit • Increases diagnostic accuracy • Requires less work • Minimizes "Oh, by the way..." at the end of the visit • Facilitates negotiating an agenda		

	Elicit the patient's concerns	• Start with open-ended questions: - "What would you like help with today?" or, - "I understand you're here for... Could you tell me more about that?" or, - What else?" • Speak directly with the patient when using an interpreter					

	Plan the visit with the patient	• Repeat concerns back to check understanding • Let patient know what to expect: "How about if we start with talking more about...then, I'll do an exam, and then we'll go over possible tests/ways to treat this? Sound OK?" • Prioritize when necessary: "Let's make sure we talk about X and Y. It sounds like you also want to make sure we cover Z. If we can't get to the other concerns, lets..."					

**Elicit the ** **patient's ** **perspective**	Ask for patient's ideas	• Assess patient's point of view: - "What do you think is causing your symptoms?" - "What worries you most about this problem?" • Ask about ideas from significant others			• Respects diversity • Allows patient to provide important diagnostic clues • Uncovers hidden concerns • Reveals use of alternative treatments or requests for tests • Improves diagnosis depression and anxiety		

	Elicit specific requests	• Determine patient's goal in seeking care: "When you've been thinking about this visit, how were you hoping I could help?"					

	Explore the impact on the patient's life	• Check context: "How has the illness affected your daily activities, work, or family?"					

**Demonstrate ** **empathy**	Be open to patient's emotions	• Assess changes in body language and voice tone • Look for opportunities to use brief empathic comments or gestures			• Adds depth and meaning to the visit • Builds trust, leading to better diagnostic information, adherence, and outcomes		

	Make at least one empathic statement	• Name a likely emotion: "That sounds really upsetting." • Compliment patients on efforts to address problem			• Makes limit-setting or saying "no" easier		

	Be aware of your own reactions	• Use own emotional response as a clue to what patient might be feeling • Take a brief break if necessary					

**Invest in the ** **end**	Deliver diagnostic information	• Frame diagnosis in terms of patient's original concerns • Test patient's comprehension • Improves adherence			• Increases potential for collaboration • Influences health outcomes • Improves adherence • Reduces return calls and visits		

	Provide education	• Explain rationale for tests and treatments • Review possible side effects and expected course of recovery • Recommend lifestyle changes • Provide written materials and refer to other sources			Encourages self care		

	Involve patient in decision making	• Discuss treatment goals • Explore options, listening for the patient's preferences • Set limits respectfully: "I can understand how getting that test makes sense to you. From my point of view, since the results won't help us diagnose or treat your symptoms, I suggest we consider this instead." • Assess patient's ability and motivation to carry out plan					

	Complete the visit	• Ask for additional questions: "What questions do you have?" • Assess satisfaction: "Did you get what you needed?" • Reassure patient of ongoing care					

^©^1996, 1999, 2003 by The Permanente Medical Group, Inc., Physician Education and Development Rao *et al. BMC Health Services Research *2010 **10**:97 doi:10.1186/1472-6963-10-97

The Four Habits Model has recently been validated and is being used in physician education and research. In particular, the model has been used to teach clinicians in different stages of training and specialties (e.g., medical students, general practitioners, geriatricians), and has been adapted to a variety of communication topics, such as end-of-life issues, cultural competence, and cost-related conversations [8,9]. Investigators have also used the Four Habits Model to evaluate the communication practices of physicians with exceptional patient satisfaction ratings [10] and assess the communication skills of physicians who practice in other countries [11].

We were interested in obtaining expert opinion on adapting the Four Habits Model to make it more clearly reflective of the patient's side of the physician-patient interaction. To begin this task, we involved international and national experts in communication research in a consensus-building process. The experts were asked, "Which patient verbal communication behaviors should be added to the Four Habits Model so that it reflects the interactive nature of communication between physicians and patients?" We describe our approach and findings and discuss the implications of our results. We also explore potential next steps to move the field of patient-physician communication research forward.

## Methods

The Delphi method was the central approach used in this study. This method was first developed in the early 1950s as a tool for setting military priorities and since then, has been used to solve a variety of problems, such as helping groups develop educational priorities, performance indicators, and treatment guidelines [12-16]. Based on the premise that pooled intelligence enhances individual judgment and captures the collective opinion of experts [12,14,15], the Delphi technique is valued for its ability to structure and organize group communication [16]. The process typically involves multiple interactions with participants who complete two or more rounds of surveys over a relatively short period of time [14,15,17,18].

**Figure 1:**
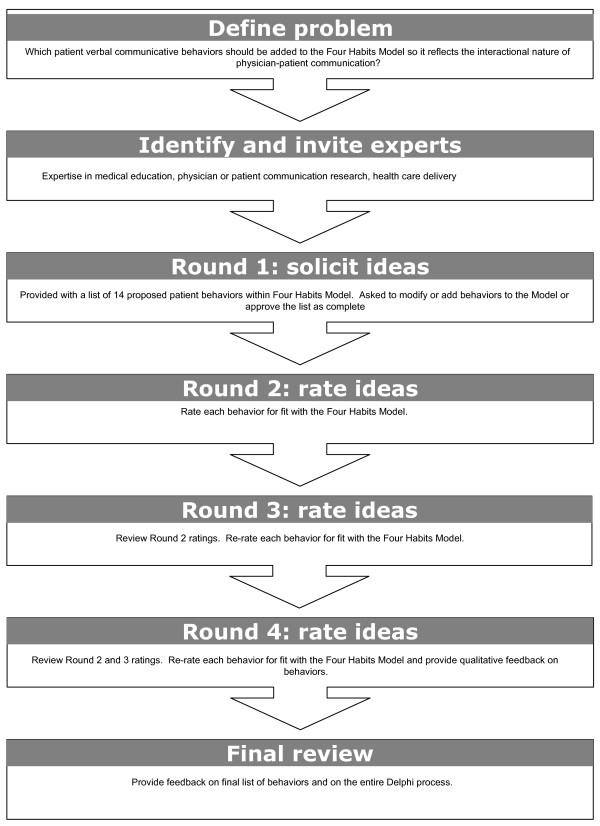
**Steps of the Delphi method used in this project**.

Although there are other methods that help facilitate consensus among experts [13,19,20], the Delphi technique's characteristics of expert anonymity and structured communication offer a number of specific advantages. The process allows experts who are in geographically-distinct locations to participate over time, and as a result, is more cost-effective (in terms of time and expense) than convening multiple face-to-face meetings [15,18,19]. Because they participate in the rounds asynchronously, the experts have an opportunity to consider the issue and provide their input without time or group pressures [14,18]. Moreover, the structured communication feature of the Delphi method helps facilitate group consensus, or score stability, while avoiding interpersonal influences [12,14,16]. Finally, this process is known to conclude with a moderate perceived sense of closure and accomplishment among participants [21]. Figure 1 illustrates the steps of the Delphi technique employed in this project. The study was reviewed and approved by the Centers for Disease Control and Prevention according to the Department of Health and Human Services Policy for Protection of Human Research Subjects.

The first phase involved nominating experts to participate in the Delphi process. Three members of the project team (JKR, LAA, RMF) identified a group of experts in patient-physician communication. The group included international researchers who have conducted patient or physician communication interventions, medical educators, and experts in health services delivery and patient-centered care (e.g., health services researchers, health care administrators). Our invitation contained information on the Four Habits Model, Delphi method, and purpose of our project. Those who accepted our invitation were informed that they were required to respond to the first round in order to participate in subsequent rounds of ratings.

Three members of the project team (JKR, BS, DB) oversaw all four rounds of the Delphi process. The entire process took place between January 15 and May 2, 2008 and was conducted using Survey Monkey, a web-based survey and data collection system. In round 1, we provided the experts with a slightly modified version of the Four Habits Model that only contained physician verbal communication behaviors (i.e., non-verbal behaviors such as "convey empathy non-verbally" were not included) and a list of proposed patient verbal communication behaviors (herein referred to as "patient behaviors") within each habit. The proposed list of patient behaviors was based on our review of communication interventions directed toward patients and consisted of the verbal communication outcomes reported in these studies [3]. The participants were reminded that our goal was to identify patient behaviors to add to the Four Habits Model so it would be more reflective of the interactional nature of physician-patient communication. They were asked to review the list of proposed patient behaviors and indicate if it was complete. If a participant thought the list was incomplete, he or she was asked to add specific behaviors to the framework. Additionally, participants could change the wording of the proposed behaviors or move behaviors between habits. With each of these options, we emphasized our focus on identifying measurable verbal communicative behaviors within the framework and asked that their suggestions meet this requirement.

In rounds 2, 3 and 4, the participants were instructed to consider the behaviors originally proposed by the project team and the behaviors they added or modified in round 1. For the originally-proposed behaviors and suggested additions, they were asked to indicate whether each behavior fit within the associated habit (agree or disagree). For behaviors with suggested modifications, the participants could choose to retain the behavior in its original form, select one of the modifications, or remove the behavior from the habit.

After each round, we calculated the percent agreement for each behavior and presented these data in the next round. We asked the participants to review this information and re-rate each behavior for its fit with the habit. The literature on the Delphi method suggests that investigators establish decision rules regarding the handling of rating information and the definition of consensus a priori [13,15,16]; defining consensus based on a specific percentage level is a common approach [16]. Thus, before this study began, we established that behaviors that received greater than 70% of the experts' votes as achieving consensus. Once this level was reached, these behaviors were not included in subsequent rounds of ratings. Behaviors that received 0 votes were also excluded from subsequent rounds (i.e., reached consensus to drop). For the suggested additions, if more than 70% of the experts agreed that a behavior fit the habit, it was retained (i.e., achieved consensus to retain); if less than 30% agreed that a behavior fit the habit, it was dropped (i.e., achieved consensus to drop). For combinations of originally-proposed behaviors with suggested modifications, only the choices that received 0 votes were dropped from the subsequent round. The remaining items were included in the next rounds until one of the options in the set received more than 70% of the experts' votes or until the entire rating process was concluded.

In round 4, we presented all behaviors that achieved consensus and rating data for the behaviors that did not achieve consensus. In this round, the experts were given a final opportunity to rate and comment on the behaviors that had not achieved consensus in round 3. After round 4, the project team collated the rating data from all rounds to develop a list of behaviors that did and did not reach consensus. The experts were invited to provide feedback on the list and the overall Delphi process.

## Results

We invited 31 international experts in patient-physician communication to participate in the Delphi process. This group included 17 physicians (training: 13 internal medicine, 3 family practice, 1 anesthesiology), 13 PhD scientists (training: 8 psychology, 2 communication research, 1 public health, 2 health services research), and 1 physician assistant. In the United States (US), a physician assistant is a health professional who is licensed to provide basic medical services under the supervision of a licensed physician. Of these individuals, 5 declined (2 internists, 2 family practitioners, 1 PhD (psychology)) and 3 PhD scientists (public health, communication research, health services research) did not respond to our invitation. Thus, 23 experts responded to our initial invitation and agreed to participate; this group was balanced with respect to gender (11 females, 12 males) and included 5 individuals from countries outside of US. In terms of educational background, 13 were physicians (11 internists, 1 family practice, 1 anesthesiologist), with 1 physician assistant and 9 PhD scientists (7 psychology, 1 communication research, 1 health services research).

Of the 23 experts, 17 responded to round 1 and were invited to participate in the subsequent rounds. Overall, this group included 11 males and 3 individuals from non-US countries and was comprised of 9 physicians (8 internists, 1 family practitioner), 7 PhDs (5 psychology, 1 health services research, 1 communication research) and 1 physician assistant. The 6 experts (4 physicians (3 internists, 1 anesthesiologist), 2 PhD (psychology)) who did not respond to round 1 were not involved in subsequent rounds.

Round 1 of the Delphi process began with 14 patient behaviors that were proposed by the project team. In this round, the 17 experts accepted 2 of the proposed behaviors, modified the remaining 12 behaviors, and added 20 new behaviors to the model (Table 2). Of the 20 suggested additions, 5 were added to Habit 1, 6 to Habit 2, 4 to Habit 3, and 5 to Habit 4. The experts proposed a total of 25 modifications to the original 12 behaviors, ranging from 1 to 4 modifications per behavior. Some of the modifications consisted of minor wording changes (i.e., "share" instead of "disclose" all relevant concerns) while other suggestions were more extensive (change "tell story" to "tell story with answers to Kleinman's explanatory model [22]"). Overall, the experts directed most suggestions to Habit 2 (12 modifications and 6 additions), with the fewest suggestions made to Habit 3 (2 modifications and 4 additions).

**Table 2:** Results from round 1 of the Delphi process

Habit	Proposed list of patient behaviors (original)	Changes suggested in Round 1
**Invest in the ** **beginning**	Make personal remarks	Modifications Respond to clinician's welcome
		Engage in social talk until comfortable
		Return the physician's greeting
	Disclose relevant concerns	Modifications
		Disclose all relevant concerns to the best of my knowledge
		Share all relevant concerns
	Articulate priorities	Modification
		State which concerns are most important
		Additions
		Approve or modify stated agenda for the visit
		Set the agenda for the visit
		Solicit physician's goals for the visit
		Explain how one learns best
		Greet or verbally welcome physicians

**Provide your ** perspective*	Express preferences	Modification Share values
	Express expectations	Modifications
		State specific requests
		Share wishes and expectations
		Express hopes, desires, expectations
	Describe impact	Modification
		Tell how illness affects daily life
	Participate in the visit	Modifications
		Provide details about symptoms, concerns, and previous treatments
		Give opinions about possible causes of illness
		Answer questions in reasonable detail, raise new comments, demonstrate respect
	Tell story	Modifications
		Offer information about medical history
		Tell the whole story of illness
		Tell the whole story of your concern(s)
		Tell story with answers to Kleinman's explanatory model
		Additions
		Explain personal beliefs or worries about symptoms
		Honestly point out when areas are left unaddressed
		State own understanding of problem (explanatory model) to the physician
		Give frank opinions about choices offered
		Offer explanations
		Ask questions about explanations and choices

**Communicate **	Express feelings and concerns	Modifications
**about your feelings **		Discuss psychosocial issues and express feelings and concerns
and concerns*		Express and elaborate on feelings and concerns
		Additions
		Explore impact of feelings and concerns on health
		Respond to physician expression of empathy
		Talk openly about psychosocial issues
		Listen to physician for understanding

**Invest in the end**	Use own language and terms	Modification
	to describe diagnosis	Use own language and terms to describe diagnosis, evaluation or treatment plans
	Participate in decision making	Modifications
		Express level of participation in decision making desired and participate to that extent
		Make opinions or concerns about choices known
		State preferences about tests or treatment options
	Ask questions	No modifications suggested
	Seek clarifying information	No modifications suggested
	Restate recommendations and information provided by doctor	Modification Restate decisions made during the visit
		Additions
		Describe any barriers to following the treatment plan
		Ask the doctor to explain any words or ideas that are confusing
		Explore barriers to implementing the plan
		Answer the "Ask me 3" questions (e.g., "What is my main problem?", "What do I need to do?", "Why is it important for me to do this?")
		Ask about options interested in but not mentioned by the doctor

*Names of Habit 2 (Elicit the patient's perspective) and Habit 3 (Demonstrate empathy) were modified to reflect the health consumer's perspective

Based on input from round 1, the 17 experts were presented with 59 behaviors in round 2 along with 14 options to remove specific behaviors from habits (Table 3). Of the behaviors presented, 10 behaviors reached consensus (i.e., 12 or more experts agreed that the behavior fit the habit) and were retained (Table 3, round 2 (column 2)). The retained behaviors were in Habits 2 (4 behaviors) or Habit 4 (6 behaviors). At the same time, 11 items were dropped (8 received 0 votes and 3 received votes from 5 or fewer experts. All 17 experts responded to round 2.

**Table 3:** Results of the Delphi ratings by round

Items after Round 1*	Round 2** (n = 17)	Round 3** (n = 16)	Round 4** (n = 16)	Final result
**Habit 1: Invest in the beginning**				

Make personal remarks (original)	1/17†	0	Drop	Drop
Respond to clinician's welcome (modification)	7/17	8/16	8/16	--
Engage in social talk until welcome (modification)	4/17	2/16	2/16	--
Return the physician's greeting (modification)	3/17	3/16	2/16	--
Remove "make personal remarks" from this habit	2/17	3/16	4/16	--

Disclose relevant concerns (original)	6/17	1/16	Drop	Drop
Disclose all relevant concerns to the best of my knowledge (modification)	2/17	2/16	Drop	Drop
Share all relevant concerns (modification)	9/17	13/16		**Retain**
Remove "disclose relevant concerns" from this habit	0	Drop		Drop

Articulate priorities (original)	9/17	6/16	2/16	Drop
State which concerns are most important (modification)	8/17	10/16	14/16	**Retain**
Remove "articulate priorities" from this habit	0	Drop		Drop

Approve or modify stated agenda (addition)	10/16	15/16		**Retain**
Set the agenda for the visit (addition)	11/17	11/16	9/16	--
Solicit the physician's goals for the visit (addition)	7/16	4/16	Drop	Drop
Explain how one learns best (addition)	3/16	Drop		Drop
Greet or verbally welcome the physician (addition)	11/16	13/16		**Retain**

**Habit 2: Provide your perspective**				

Express preferences (original)	14/17			**Retain**
Share values (modification)	2/17	Drop		Drop
Remove "express preferences" from this habit	1/17			Drop

Express expectations (original)	5/17	5/16	9/16	--
State specific requests (modification)	2/17	1/16	1/16	--
Share wishes and expectations (modification)	5/17	5/16	2/16	--
Express hopes, desires, and expectations (modification)	5/17	5/16	4/16	--
Remove "express expectations" from this habit	0			Drop

Describe impact (original)	2/17			Drop
Tell how illness affects daily life (modification)	15/17			**Retain**
Remove "describe impact" from this habit	0			Drop

Participate in visit (original)	6/17	7/16	7/16	--
Provide details about symptoms, concerns, and previous treatments (modification)	3/17	6/16	4/16	--
Give opinions about possible causes of illness (modification)	0			Drop
Answer questions in reasonable detail, raise new comments, demonstrate respect (modification)	3/17	0		Drop
Remove "participate in visit" from this habit	5/17	3/16	5/16	--

Tell story (original)	7/17	8/16	12/16	**Retain**
Offer information about medical history (modification)	1/17	0		Drop
Tell the whole story of illness (modification)	3/17	2/16	0	Drop
Tell the whole story of your concern(s) (modification)	3/17	3/16	0	Drop
Tell story with answers to Kleinman's explanatory model questions (modification)	2/17	2/16	1/16	Drop
Remove "tell story" from this habit	1/17	1/16	3/16	Drop

Explain personal beliefs or worries about symptoms (addition)	15/15			**Retain**
Honestly point out when areas are left unaddressed (addition)	7/15	11/16	15/16	**Retain**
State own understanding of problem (explanatory model) with physician (addition)	11/17	14/16		**Retain**
Give frank opinions about choices offered (addition)	9/16	15/16		**Retain**
Offer explanations (addition)	3/15			Drop
Ask questions about explanations and choices (addition)	13/17			**Retain**

**Habit 3: Communicate about your feelings and concerns**				

Express feelings and concerns (original)	8/17	13/16		**Retain**
Discuss psychosocial issues and express feelings and concerns (modification)	3/17	0		Drop
Express and elaborate on feelings and concerns (modification)	6/17	3/16		Drop
Remove "express feelings and concerns" from this habit	0			Drop

Explore impact of feelings and concerns on health (addition)	5/16	7/16	9/16	--
Respond to physician's expression of empathy (addition)	5/15	4/16		Drop
Talk openly about psychosocial issues (addition)	7/16	13/16		**Retain**
Listen to physician for understanding (addition)	4/16			Drop

**Habit 4: Invest in the end**				

Use own language and terms to describe diagnosis (original)	1/17			Drop
Use own language and terms to describe diagnosis, evaluation or treatment plans (modification)	12/17			**Retain**
Remove "use own language and terms to describe diagnosis" from this habit	4/17			Drop

Participate in decision making (original)	3/17	4/16	2/16	--
Express level of participation in decision making desired and participate to that extent (modification)	8/17	10/16	11/16	--
Make opinions or concerns about choices known (modification)	2/17	1/16	1/16	--
State preferences about tests or treatment options (modification)	4/17	1/16	2/16	--
Remove "participate in decision making" from this habit	0			Drop

Restate recommendations and information provided by doctor (original)	8/17	9/16	13/16	**Retain**
Restate decisions made during the visit (modification)	8/17	5/16	2/16	Drop
Remove "restate recommendations and information provided by doctor" from this habit	1/17	2/16	1/16	Drop

Ask questions (original)	17/17			**Retain**
Remove "ask questions" from this habit	0			Drop

Seek clarifying information (original)	16/17			**Retain**
Remove "seek clarifying information" from this habit	1/17			Drop

Describe any barriers to following the treatment plan (addition)	14/16			**Retain**
Ask the doctor to explain any words or ideas that are confusing (addition)	13/17			**Retain**
Explore barriers to implementing the plan (addition)	8/17	12/16		**Retain**
Answer the "Ask me 3" questions (addition)	7/17	7/16	15/16	Drop
Ask about options interested in but not mentioned by the doctor (addition)	13/17			**Retain**

*Original behaviors were proposed by the project team. During Round 1 the experts suggested modifications to the original behaviors and behaviors that should be added (i.e., additions) to the Model.

**17 experts participated in Round 2, and 16 in Rounds 3 and 4.

†In each round, the experts rated each behavior (agree or disagree) for their fit with a particular habit; each column includes the percent agreement received by each behavior. Behaviors that received more than 70% of the experts votes (either disagree or agree) were defined as achieving consensus (either retain or drop). Behaviors that received 0 votes were not included in subsequent rounds or ratings.

In Round 3, the 17 experts were presented with 42 behaviors and 4 options to remove specific behaviors, and they were asked to re-rate these items. Another 8 behaviors (i.e., 6 additions, 1 modification, and 1 originally-proposed behavior) received 12 or more of the experts' votes and were retained (Table 3, round 3 (column 3)). Six behaviors were dropped (2 received votes from 4 or fewer experts and 4 received 0 votes). Additionally, 3 behaviors were dropped at this point because they were part of a set in which a related behavior reached consensus. Sixteen of the 17 experts responded to round 3; 1 physician (an internist) did not respond to this round.

In Round 4, the 17 experts were asked to rate the remaining 25 behaviors and 4 remove options. At this time, an additional 4 behaviors (2 original, 1 modification, 1 addition) received 12 or more of the experts' votes and were retained while 6 behaviors and 2 remove options were dropped (Table 3, round 4 (column 4)). During this round, the experts also had an opportunity to provide feedback on the 25 behaviors being rated. Some commented that there were overlaps between the behaviors they were rating with behaviors that had already reached consensus in prior rounds or certain habits already contained too many consensus behaviors. Other experts expressed concerns with the wording of specific choices or that a particular behavior (for example, "explore impact of feelings on health") would place a significant burden on the patient or might represent an unrealistic expectation for typical patients. Sixteen of the 17 experts responded to round 3; 1 physician (an internist) did not respond to this round.

After 3 rounds of rating, the experts came to consensus on 22 behaviors to retain in the framework (Table 3, final results (column 5)). These behaviors were distributed across the habits, with 4 behaviors in Habit 1, 8 in Habit 2; 2 in Habit 3, and 8 in Habit 4. At the same time, 34 behaviors were dropped and consensus was not reached for 17 items. The project team reviewed and made minor revisions to the retained behaviors that reached consensus and to the names of the habits so they reflected the patients' perspective. The list of patient behaviors was distributed to the experts for their review and feedback. Of the 12 experts who provided feedback, nearly all stated that they were comfortable with the final list of patient behaviors and with the Delphi process overall. Two experts expressed concerns about not including "participate in decision making" in the final list; this was among the behaviors that did not reach consensus. Table 4 presents a proposed framework that includes patient habits and communication behaviors (columns 1 and 2) derived from our consensus process as well as physician habits and communication behaviors (columns 3 and 4) from the original Four Habits Model.

**Table 4:** Patient communication behaviors identified through expert consensus and physician communication behaviors within the Four Habits Model Framework

← Consensus Behaviors →		← Original Four Habits Model →	
**Patient Habit**	**Patient behaviors**	**Physician Habit**	**Physician behaviors**

**Invest in the beginning**	Greet or verbally welcome health care provider Share all relevant concerns State which concerns are most important Approve or modify agenda	**Invest in the beginning**	Create rapport quickly Elicit the patient's concerns Plan the visit with the patient

			

**Provide your perspective**	Share your story State own understanding of problem Tell how illness affects daily life Explain personal beliefs or worries about symptoms Express preferences (such as desires for specific tests, treatment, decision making)	**Elicit the patient's perspective**	Ask for the patient's ideas Elicit specific requests Explore the impact on the patient's life

			

**Communicate about your feelings and concerns**	Express feelings and concerns Talk openly about psychosocial issues (such as anxiety, fear, sadness) Indicate if areas have not been addressed Ask healthcare provider to explain any words or ideas that are confusing	**Demonstrate empathy**	Be open to patient's emotions Make at least one empathic statement Convey empathy nonverbally Be aware of your own reactions

			

**Invest in the end**	Use own words to summarize information and recommendations provided by the health care provider Ask questions about explanations and choices Give frank opinions about choices offered Describe or explore any barriers to following recommendations Ask about options interested in but not mentioned by the health care provider Conclude visit	**Invest in the end**	Deliver diagnostic information Provide education Involve patient in decision making Complete the visit

Columns 1 and 2 represent patient behaviors identified in the consensus process involving experts in communication research

Columns 3 and 4 represent the original Four Habits Model (Stein, Frankel, Krupat)

## Discussion

Although several investigators have described the physician [6,23,24] and patient [24-26] communication behaviors that are desirable elements of patient-centered communication, the literature lacks a generally-accepted model for how both sets of behaviors might unfold and influence the course and outcome of a clinical encounter. An overarching model of patient-physician communication might prompt researchers to examine the degree to which interventions directed toward one party (i.e., either physicians or patients) influence the communication behaviors of the interaction partner. Moreover, a framework that describes the sequence of communication behaviors could help guide interventions directed toward specific segments of the interaction. The Four Habits Model has been particularly useful in describing the appropriate physician communication behaviors during the course of a clinical interaction and serving as a research framework for interventions promoting patient-centered communication skills among physicians [9]. We believed that this Model could serve as a useful starting point for developing a framework that includes both physician *and *patient communication behaviors. As a first step in this direction, we conducted a consensus process involving a group of communication experts to identify an initial set of patient communication behaviors that could affect the course, direction and outcomes of the medical encounter.

Seventeen international experts in communication research, medical education and health care delivery participated in the consensus process. We found the Delphi method useful in soliciting input and building consensus within this group. The experts were quite engaged throughout the entire process, as evidenced by the numerous suggestions (25 modifications, 20 additions) they made in round 1 and the greater than 90% response rate to all three rounds of ratings. Most of their suggestions pertained to Habit 2 ("Provide your perspective") with Habit 3 ("Communicate your feelings and concerns") receiving the least number of suggestions.

After three rounds of ratings, the experts agreed to retain 22 behaviors which were distributed throughout the Model. Interestingly, 6 behaviors that the experts agreed to retain were among the 14 originally-proposed by the project team. The final list addresses key aspects of patient communication, such as sharing and prioritizing concerns, expressing feelings, and summarizing information and recommendations provided by the physician. While most behaviors can be assessed using existing coding schemes, the list also includes a few behaviors that may require methods that examine the content of statements made by the patient. Examples of such behaviors include "describe how the illness affects (one's) daily life" and "share your story".

Of the behaviors that the experts agreed to drop or which ultimately did not achieve consensus (in either direction), most were similar to other behaviors that were retained. For example, "give opinions about possible causes of (my) illness," a suggestion that received 0 votes, is comparable to "state own understanding of the problem," a retained behavior. Similarly, "respond to the clinician's welcome," which did not achieve consensus is comparable to the retained behavior, "greet or verbally welcome the physician." Several experts also noted that certain behaviors were not practical for the typical patient (e.g., "solicit the physician's goals for the visit"), which may explain why they were dropped from the Model.

The experts were unable to reach consensus on whether or not to retain "participate in decision-making" or any of its suggested modifications. Interestingly, the literature lacks a detailed definition of what constitutes patient participation or involvement in decision making [27,28]. In a recent study of physician-patient communication related to breast cancer decisions, Brown and colleagues [29] assessed whether the patient: presented her agenda; declared her preferences (for information and involvement); declared her perspectives (costs and benefits of treatment); and portrayed herself in an active role. To a certain extent, these elements were retained, even though they are not labeled as participation in decision-making. For example, Habit 2 includes: "express preferences...". Similarly, Habit 4 includes: "use own words to summarize information...," "give frank opinions about the choices offered," "ask questions about explanations and choices," "describe any barriers to following recommendations," and "ask about options interested in but not mentioned by the health care provider".

The following limitations should be considered when reviewing our findings. First, our project focused on verbal communication behaviors only because we believe that these behaviors might serve as a logical starting point for developing educational initiatives for patients. Second, our results should not be interpreted as representing all the views of experts in the field of communication, particularly patient communication. Our panel included those with expertise in developing interventions to enhance physician communication as well as those who focus specifically on improving patient communication skills. We chose experts with diverse interests to acknowledge the interactive nature of physician-patient communication (i.e., to recognize that the physician's communication behaviors influences the patients' communication behaviors).

Third, it is important to note that the Four Habits Model describes a set of basic physician communication behaviors for the clinical encounter. Studies of the medical interview which were conducted in primary care with adult patients, other conceptual models of communication and consensus statements (i.e., Kalamazoo consensus statement) informed the development of the Four Habits Model [9,30]. Thus, the original model and patient behaviors suggested through our consensus process may not apply to certain situations, such as communication between physicians and caregivers, pediatric patients, or psychiatric patients. Likewise, the Four Habits Model and our consensus behaviors do not reflect the potential differences in communication styles due to the gender or ethnic background of the physician and/or patient. Additional research is necessary to examine (and adapt) the Four Habits Model and the consensus behaviors to these other circumstances.

Finally, our consensus process involved professionals, and we chose this approach for several reasons. Because of our interest in identifying measureable communication behaviors for an overarching conceptual framework, we believed that experts who were familiar with the communication research and education literature would be helpful in generating an initial list in an efficient manner. We also believed that developing the list of patient behaviors was a necessary step for guiding our subsequent work with patients. All 17 experts who participated in the consensus process have years of experience working to enhance the patient's experience of health care, and it is noteworthy that 7 members of the panel were behavioral scientists and not physicians. The fact that the group eliminated behaviors that are not practical for the average patient is a demonstration of their commitment to the patient's perspective.

We recognize that starting with expert opinion could potentially bias the subsequent modification of the communication skills identified through this process and is an important limitation of our study. We also acknowledge that our approach may appear to "privilege" the experts' voice in determining what communication skills and behaviors patients might find useful in communicating with their physicians. By the same token, however, one could argue that a similar bias would exist had we conducted a Delphi process with patients only, not to mention the difficulty that would be entailed in selecting different "types" of patients to participate in such a process. We firmly believe that patients *should *be involved in the validation process, and this process should involve patient populations: with different demographic characteristics (i.e., gender, race/ethnicity); who are vulnerable (e.g., limited health literacy, older adults, lower socioeconomic status); or with varying degrees of medical co-morbidity (i.e., relatively healthy versus relatively complex). Potential approaches could include focus groups or semi-structured interviews with patients, analogous to previous studies which examined patients' perspectives regarding informed decision making [31-33]. In addition, the patient behaviors should be validated using audiotapes of actual encounters, an approach that was used to validate the physician behaviors within the Four Habits Model [8].

## Conclusion

Although much progress has been made in improving physician-patient communication, researchers in the field have tended to focus on either physicians or patients and not consider the interactive nature of communication. This approach may relate to the lack of an overarching model of physician-patient communication that describes how both sets of behaviors unfold and affect the course and outcome of an interaction. The Four Habits Model is a teaching and research framework that describes the sequence of important physician communication behaviors during the outpatient encounter. Our consensus process involving 17 international experts identified 22 patient verbal communication behaviors which would add the patient's voice to this model. We believe that integrating patient behaviors into the current Four Habits framework is an important step in creating a research and education agenda that could improve communication on both sides of the stethoscope.

## Competing interests

The authors declare that they have no competing interests.

## Authors' contributions

JKR, LAA, RMF, and BS conceptualized and contributed to the design of this study. JKR worked with BS and DB to collect the data. JKR, LAA, RMF, BS, and TS participated in the analysis and interpretation of the findings. JKR and LAA wrote the first draft of the manuscript, and all authors reviewed and revised drafts of the manuscript. All authors read and approved the final manuscript.

## Funding information

Dr Sukumar and Ms. Beauchesne were supported through a contract between the Centers for Disease Control and Prevention and ICF Macro International (Contract Number: 200-2007-M-21989). Drs Rao, Anderson, Stein, and Frankel did not have a financial relationship with ICF Macro International.

## Pre-publication history

The pre-publication history for this paper can be accessed here:

http://www.biomedcentral.com/1472-6963/10/97/prepub
